# Hospitalized Women's Willingness to Pay for Inpatient Screening Colonoscopy

**DOI:** 10.1089/whr.2022.0014

**Published:** 2022-09-13

**Authors:** Opeoluwa Olayinka, Jerome Gnanaraj, Waseem Khaliq

**Affiliations:** Department of Medicine, Johns Hopkins Bayview Medical Center, Johns Hopkins University School of Medicine, Baltimore, Maryland, USA.

**Keywords:** willingness to pay, inpatient screening colonoscopy, hospitalized women

## Abstract

**Background:**

Despite the proven mortality benefit of screening colonoscopy, ∼27% of hospitalized women are nonadherent with colorectal cancer (CRC) screening guidelines. Colonoscopy is the most frequently used test for CRC screening in the United States. Although CRC is the second most common cause of cancer death in the United States, CRC screening has not been part of usual hospital care.

**Objective:**

This study explores how hospitalized women perceive value of inpatient screening colonoscopy by evaluating the mean amount of money that hospitalized women are willing to contribute toward the cost of a screening colonoscopy during a hospital stay.

**Methods:**

A cross-sectional bedside survey consisting of a contingent valuation questionnaire was used to assess the contribution these women considered to be justified for the convenience of an inpatient screening colonoscopy. The probit regression model was used for the analysis of contingent valuation data to predict mean willingness to pay toward inpatient screening colonoscopy.

**Results:**

Of the 312 enrolled patients, 48% were willing to pay a mean of $171.56 (95% confidence interval [CI] $37.59–$305.54, *p* = 0.012) in advance toward the cost of an inpatient screening colonoscopy. After adjustment of possible sociodemographic and clinical covariates that could impact willingness to contribute, hospitalized women were willing to pay a mean of $178.41 (95% CI $40.67–$316.16, *p* = 0.011).

**Conclusions:**

The findings of this study suggest that hospitalized women value the prospect of screening colonoscopy during hospitalization. Offering screening colonoscopy to nonadherent hospitalized women, especially those who are at high risk for developing CRC, may improve adherence among hospitalized women.

This study is registered at www.clinicaltrials.gov (NCT04162925).

## Introduction

Colorectal cancer (CRC) is the second most common cause of cancer death in the United States.^1^ The lifetime risk of developing CRC among women is 1 in 25 (4.1%).^2^ The U.S. Preventive Services Task Force (USPSTF) recommends that all individuals aged 50–75 years undergo CRC screening *via* any stool-based or direct visualization tests.^3^ Colonoscopy every 10 years is among the most frequently used CRC screening test in the United States and among the most effective in reducing CRC mortality.^4^

A U.S. population-based survey indicates that current CRC screening rates among women aged 50–75 years are 66%.^5^ Despite national campaigns to improve CRC screening, significant barriers persist. These include: screening awareness, perception of the importance of screening, education, socioeconomic status, lack of insurance, transportation issues, lack of counseling by primary care providers, and out of pocket cost.^6,7^

A recent meta-analysis reported that adherence to CRC screening remains low among women in general compared with men.^8^ The gender disparity persists even after adjustment for income, education, and access to health insurance coverage.^8^ A recent study evaluating the prevalence of nonadherence to CRC screening guidelines reported that ∼27% of hospitalized women aged 50–75 years are overdue for CRC screening and 9% were at high risk for developing CRC.^7^ Almost two thirds of nonadherent women in that study would agree to have an inpatient screening colonoscopy if it was due and offered during a hospital stay.^7^

CRC screening *via* colonoscopy is usually offered in outpatient settings, and inpatient screening colonoscopy rate is very low.^9^ This may be due to the challenges associated with bowel preparation or uncertainty about screening coverage by health insurance during a hospital stay, as the procedure can potentially increase the length of hospital stay and out-of-pocket expense, respectively. Due to this potential cost differential, it becomes essential to evaluate how hospitalized women, who are amenable to inpatient screening, value the potential opportunity for an inpatient screening colonoscopy and willing to contribute toward the additional cost associated with inpatient screening colonoscopy. This study further seeks to establish the amount that hospitalized women are willing to pay toward an inpatient screening colonoscopy.

## Methods

### Study design and sample

Detailed enrollment methods have been published.^7^ Briefly, we surveyed 510 women hospitalized in the internal medicine service at an academic medical center between December 1, 2014, and May 31, 2017. These women were aged 50–75 years and were cancer free at baseline. The study only included women for participation because it has been reported that women perceive CRC as a “male disease.”^8^ Thus, perceived CRC risk among women is lower compared with breast and cervical cancers. This perception is thought to be associated with both lack of awareness and lack of counseling by primary care providers.^8^ Of the enrolled women, 198 (39%) were not interested in inpatient screening colonoscopy and thus excluded from contingent valuation questionnaire.

The remaining 312 (61%) women were amenable to inpatient screening colonoscopy and thus underwent a contingent valuation questionnaire to determine the financial contribution these women considered acceptable for the convenience of an inpatient screening colonoscopy. Contingent valuation is a method used in health economics to estimate the value placed by patients for a health care service by inquiring amount the patients would be willing to pay for the service.^10^ The willingness to pay (WTP) variable may approximate patient-centeredness by allowing scientific evaluation to estimate how individuals value health care services.^11^

### Protocol and measures

A bedside survey was conducted by the study coordinator to collect data consisting of sociodemographic, CRC adherence and risk, and medical comorbidities. We asked the following contingency question: “Screening colonoscopy is recommended by U.S. Preventive Services Task Force and American Cancer Society for colorectal cancer screening every 10 years. Current practice is not to order colorectal cancer screening during one's hospital stay, and you will not have one during this admission. However, in the future, if your hospital providers were able to arrange for a screening colonoscopy while you were in the hospital, would you be willing to pay $*x* out of pocket to offset the costs of this test?”

Each respondent was randomly assigned one of seven possible amounts for *x* (*x* = $50, $75, $100, $150, $250, $500, or $1,000). The respondents were instructed to answer either “Yes” or “No,” thereby indicating whether the stated dollar amount was acceptable. All study participants provide their informed consent for participation. Institutional review board at the medical center approved study ethics and protocol.

### Statistical methods

Respondent characteristics are presented as proportions and means. Unpaired *t*-test and chi-square test were used to compare women WTP and not WTP toward inpatient screening colonoscopy. *t*-Test and chi-square test determined significance at *p*-value ≤0.05. The probit regression model was used for the analysis of contingent valuation data to predict the mean WTP.^12^ The data were analyzed using Stata, Version 13.1.

## Results

**FIG. 1.**
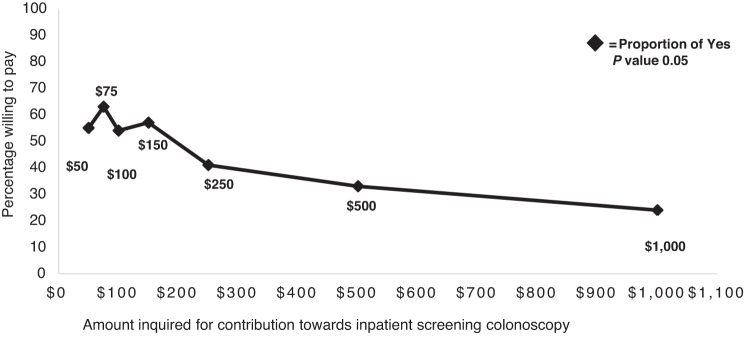
Proportion of hospitalized women willing to pay for seven amounts for inpatient screening colonoscopy.

Study participants' baseline characteristics including sociodemographic, health access and behavior, and medical comorbidity burden by WTP status can be seen in Table 1. There were no differences noted between the two groups except that women WTP were less likely to be current smokers (Table 1). There was also an inverse relation noted between proportion of women willing to contribute toward inpatient screening colonoscopy and the inquired dollar amount (*p* 0.05) in the contingent value question (Fig. 1). The median acceptable WTP amount was $100. The unadjusted mean amount hospitalized women were WTP was $171.56 (95% confidence interval [CI] $37.59–$305.54) determined using the probit regression model. After adjusting for 18 variables that could potentially influence WTP (Supplementary Appendix), the mean WTP amount was essentially unchanged ($178.41; 95% CI $40.67–$316.16).

**Table 1.** Characteristics of Study Population by Willing to Pay Inpatient Screening Colonoscopy

Characteristic^a^	Not willing to pay toward colonoscopy cost (***n*** = 161)	Willing to pay toward colonoscopy cost (***n*** = 151)	***p*** ^b^
Age, years, mean (SD)	60.6 (6.9)	59.9 (6.9)	0.36
Race, *n* (%)			
Caucasian	89 (55)	84 (56)	0.76
African American	64 (40)	62 (41)	
Others	8 (5)	5 (3)	
Married, *n* (%)	36 (22)	42 (28)	0.27
High school or more years of education, *n* (%)	130 (81)	120 (79)	0.78
Employment status, *n* (%)			0.39
Employed	32 (20)	29 (19)	
Unemployed	8 (5)	13 (9)	
Retired	48 (30)	35 (23)	
Disability/unable to work	73 (45)	74 (49)	
Ambulatory function, *n* (%)			0.55
Ambulate without assistance	90 (56)	93 (62)	
Ambulate with cane or walker	62 (38)	52 (34)	
Chronic disable, wheelchair, or bedbound	9 (6)	6 (4)	
Annual household income <$20,000, *n* (%)	81 (51)	73 (49)	0.73
Uninsured, *n* (%)	0 (0)	1 (1)	0.7
No primary care physician, *n* (%)	17 (11)	16 (11)	1.00
Presenting to hospital from home, *n* (%)	158 (98)	148 (98)	0.20
Admitted as observation, *n* (%)	16 (10)	10 (7)	0.31
Principle diagnosis by system at admission, *n* (%)			0.99
General internal medicine	50 (31)	44 (29)	
Cardiovascular	27 (17)	25 (17)	
Pulmonary	29 (18)	29 (19)	
Gastrointestinal	22 (14)	20 (13)	
Neurology	1 (0.5)	1 (1)	
Nephrology	14 (9)	10 (6)	
Oncology	1 (0.5)	2 (1)	
Rheumatology	5 (3)	7 (5)	
Psychiatry	1 (0.5)	1 (1)	
Infectious disease	8 (5)	7 (5)	
Others	3 (2)	5 (3)	
Discharge from hospital to home, *n* (%)	156 (97)	146 (97)	0.19
Nonadherent to CRC screening, *n* (%)	34 (21)	38 (25)	0.40
Never had screening colonoscopy, *n* (%)	40 (25)	48 (32)	0.21
High risk for CRC^c^, *n* (%)	15 (9)	15 (10)	1.00
First-degree relative with colon cancer^d^, *n* (%)	23 (15)	22 (15)	1.00
Length of stay, days, mean (SD)	5.5 (7.6)	4.4 (3.1)	0.09
BMI, mean (SD)	32.4 (11)	34.6 (11)	0.09
Current smoker, *n* (%)	61 (38)	40 (27)	**0.04**
Alcohol use, *n* (%)	38 (24)	34 (23)	0.89
Age-adjusted CCI >3^e^, *n* (%)	72 (45)	59 (39)	0.31
Total comorbidities (excluding CCI)^f^, mean (SD)	3.3 (1.7)	3.3 (2)	0.93

The bold represents statistical significance.

^a^ For some patients, the variables had missing value.

^b^ Chi-square (Yates-corrected *p*-value where at least 20% of frequencies were <5) and unpaired *t*-test statistic.

^c^ History of Lynch syndrome, familial adenomatous polyposis, or inflammatory bowel disease.

^d^ Family history for first-degree relative with CRC.

^e^ CCI scores of 0, 1, 2, and 3 predicting 10-year survival rates of 93%, 73%, 52%, and 45%, respectively.

^f^ Comorbidities excluded diseases accounted for CCI and include hypertension, heart disease, hypercholesterolemia, atrial fibrillation, history of pulmonary embolism or deep venous thrombosis, obstructive sleep apnea, osteoporosis, depression, chronic hepatitis, parkinsonism, hypothyroidism, nephrolithiasis, and anemia.

BMI, body mass index; CCI, Charlson Comorbidity Index; CRC, colorectal cancer; SD, standard deviation.

## Discussion

Our study shows that almost half of hospitalized women interested in inpatient screening colonoscopy were willing to pay out of pocket to help offset any extra cost associated with inpatient screening colonoscopy. This is the first study that explores perceived value of inpatient CRC screening among hospitalized women and demonstrates their reception/willingness to undergo inpatient screening. Our study did not show any significant difference in the sociodemographic status, access to health care, and medical comorbidity burden between those willing to pay and not willing to pay.

However, we noticed that a higher percentage of smokers were not willing to pay for inpatient colonoscopy. The later finding may reflect how attitudes toward self-wellness can contribute toward disease prevention. Also, the rates of nonadherence to CRC screening and the percentage of women who never had a screening colonoscopy were similar in both the groups. This indicates that the WTP for inpatient screening colonoscopy is not dependent on prior adherence to CRC screening.

Even though CRC is one of the leading causes of cancer deaths, the screening rate is still low.^1^ Minorities, rural population, uninsured, and those with Medicaid-only insurance have lower rates of screening compared with others.^13^ Based on our review of the literature, the only study evaluating inpatient screening colonoscopy rates among hospitalized patients used the National Inpatient Sample database from 2005 to 2014. This study reported five screening colonoscopies per 100,000 hospitalizations and showed that women were more likely to receive inpatient screening colonoscopy compared with men,^9^ thus concurring with our results that women are receptive and value CRC screening during an inpatient stay. In United States, CRC screening is usually an opportunistic screening that results from an office space interaction between a health care provider and patient.^14^

Some of the barriers that have been cited by hospitalized women for CRC screening include lack of awareness regarding available screening modalities, lack of transportation, lack of counseling from the primary care providers, and presence of more compelling health or other problems faced by patients.^6,7,15^ In a recent study, it has been reported that hospitalized women welcome the idea of having their hospital provider discuss CRC screening with them during a hospital stay.^7^ Moreover, 66% of hospitalized women who were nonadherent to CRC screening guidelines would agree to have an inpatient screening colonoscopy if due and offered during a hospital stay.^7^ Although the hospital admissions are stressful events for patients, there is an opportunity to capture this vulnerable population as patients reflect upon their health in general and are receptive to medical advice.^16^ This time spent in the hospital can be utilized to promote a change in their health behaviors and address their concerns or fears about cancer screening tests.^16^

Multilevel screening strategies are warranted to address the barriers affecting CRC screening adherence and exploring this missed opportunity to counsel patients and engage in discussion about CRC screening could help improve CRC screening especially in hospitalized women.^17,18^ Similar studies evaluating breast cancer screening have demonstrated that majority of hospitalized women were willing to have inpatient screening mammogram if offered during hospital stay.^16,19^ As screening colonoscopy procedure is usually performed in the outpatient setting, anticipated additional cost associated with inpatient screening colonoscopy would be likely from facility charge, type of sedation required during the procedure, polyp removal, and possible increase in the length of stay in the hospital.

Our study has several limitations. First, the study was conducted in a single hospital and might be difficult to extrapolate to the rest of the population. Second, our study included only women; however, this can also be counted as a strength of the study considering that women perceive CRC risk differently compared with men and are more likely to be nonadherent.^6,8^ Third, the study involved surveys using hypothetical questions and the reliability could be variable. However, studies have shown that hospitalized women not only value the opportunity to be screened for cancers but when offered will undergo inpatient screening.^19,20^

Fourth, potential hospital-related logistical challenges were neither ascertained nor studied, including hospital length of stay and perspective of physicians, nurses, and other support staff to complete inpatient screening colonoscopy. Fifth, we also did not evaluate gastroenterologists' perspective and hospital capacity to have routine screening colonoscopy as traditionally these facilities have urgent/emergent colonoscopies on the schedule. Sixth, we also did not evaluate patients' hospitalization-related conditions (*e.g.*, diverticulosis, colitis, inflammatory bowel disease flare) that make screening colonoscopy contraindicated. Finally, we did not explore health insurance and out-of-pocket co-pays related to inpatient screening colonoscopy, namely facility charges and physician fee structure to perform screening colonoscopy in hospital setting.

Medicare reimbursement for screening colonoscopy is reported to be $981 (includes both physician fee $188 and facility fees $793) for the hospital outpatient departments.^21^ Under Medicare's procedure price lookup tool, patients will pay $37 (20% of the Medicare-approved physician's services) out-of-pocket costs for hospital outpatient departments, as well as co-payment if the physician performs the procedure (polypectomy) in a hospital setting.^22^ Additionally, patient will pay another $159 as 20% of the facility fee on top of physician fee mentioned above. The Affordable Care Act requires that insurance policies cover certain preventative services, such as colonoscopies, at no cost to the patient.^23^

Thus, hypothetically inpatient screening colonoscopy should be covered by both Medicare and private insurance. However, it is difficult without a feasibility study to determine the actual true cost of inpatient screening colonoscopy as the total out-of-pocket cost will depend on the clinical setting, anesthesia type, geographic location, and tissue sampling during procedure. Whether the mean WTP amount by hospitalized women will cover the out-of-pocket cost for inpatient screening colonoscopy remains unknown.

Our study indicates that a significant proportion of hospitalized women are willing to get an inpatient screening colonoscopy, and more than half of them are willing to pay additional out-of-pocket cost if necessary. Offering inpatient screening colonoscopy could potentially capture a significant proportion of patients who are nonadherent to CRC screening, and hence, further studies to determine the feasibility and the cost/insurance coverage related to inpatient screening colonoscopy are warranted.

## Supplementary Material

Supplemental data

## Abbreviations Used

BMI: body mass index
CCI: Charlson Comorbidity Index
CI: confidence interval
CRC: colorectal cancer
CRIG: Collaborative Research Interest Group
SD: standard deviation
USPSTF: U.S. Preventive Services Task Force
WTP: willingness to pay
