# Supplementary material for: Hospitalized Women's Willingness to Pay for Inpatient Screening Colonoscopy
Source: Womens Health Rep (New Rochelle). 2022 Sep 13;3(1):768–73. doi: 10.1089/whr.2022.0014 (PMC9518799; doi:10.1089/whr.2022.0014)
Supplement: Supplemental data [file Suppl_Appendix.docx]

**Appendix A: The unadjusted and fully adjusted (for explanatory variables) Probit models of WTP for inpatient screening colonoscopy**

|  | **Un-adjusted probit model (n=312)** | | | **Fully adjusted probit model (n=306)** | | |
| --- | --- | --- | --- | --- | --- | --- |
| **Variable** | **Beta-coeff.** | **SE of coeff.** | **p-value** | **Beta-coeff** | **SE of coeff.** | **p-value** |
| WTP | -.0010606 | .0003267 | 0.001 | -.0011085 | .0003556 | 0.002 |
| Age |  |  |  | -.2925124 | .1788287 | 0.102 |
| Race |  |  |  | -.1483316 | .160497 | 0.355 |
| Marital status |  |  |  | .0667458 | .189864 | 0.725 |
| Education |  |  |  | -.0801701 | .1969594 | 0.684 |
| Employment |  |  |  | .1773382 | .215312 | 0.410 |
| Annual household income |  |  |  | -.0529954 | .1756552 | 0.763 |
| No Primary care provider |  |  |  | .1139252 | .2538418 | 0.654 |
| Ambulatory status |  |  |  | -.1574768 | .1650003 | 0.340 |
| Hospitalized under observation |  |  |  | -.2924653 | .2913824 | 0.316 |
| Length of stay in hospital |  |  |  | -.0331397 | .0198991 | 0.096 |
| Obesity |  |  |  | .1051209 | .1575998 | 0.505 |
| Non-adherence to colorectal cancer screening |  |  |  | .0813242 | .1887909 | 0.667 |
| Current smoking |  |  |  | -.5063275 | .1792399 | 0.005 |
| Alcohol use |  |  |  | -.091664 | .1886159 | 0.627 |
| High risk for colorectal cancer |  |  |  | .0695598 | .2656927 | 0.793 |
| Family history colorectal cancer |  |  |  | .0963476 | .214752 | 0.654 |
| Charlson comorbidity index (CCI) |  |  |  | -.0454803 | .1776075 | 0.798 |
| Total comorbidities excluding CCI |  |  |  | -.0249078 | .169727 | 0.883 |
| Constant | .181962 | .0978379 | 0.063 | .6753898 | .3448153 | 0.050 |

Un-adjusted model: LR chi2 (1) = 11.20, Prob > chi2 = 0.0008

Fully adjusted model: LR chi2 (19) = 29.73, Prob > chi2 = 0.0554
